# 4D flow-sensitive MR estimation of pulmonary vascular resistance

**DOI:** 10.1186/1532-429X-15-S1-P128

**Published:** 2013-01-30

**Authors:** Alejandro Roldán-Alzate, Christopher Francois, Oliver Wieben, Naomi C Chesler, Alex Frydrychowicz

**Affiliations:** 1 Radiology, University of Wisconsin, Madison, WI, USA; 2 Biomedical Engineering, University of Wisconsin, Madison, WI, USA; 3 Medical Physics, University of Wisconsin, Madison, WI, USA; 4 Clinic for Radiology and Nuclear Medicine, University Hospital Schleswig-Holstein, Lübeck, Germany

## Background

Pulmonary arterial hypertension (PAH) is characterized by progressive increase in pulmonary vascular resistance (PVR), leading to right ventricular (RV) failure [1]. PVR is calculated using right heart catheterization (RHC), from the trans-pulmonary pressure gradient (ΔP) and pulmonary flow (QPA). Doppler echocardiography can estimate PVR from the ratio of peak tricuspid regurgitation velocity (TRV) to main pulmonary artery velocity time-integral (MPA flow) [2]. MRI is increasingly used to assess right ventricular (RV) function in PAH. Determining PVR from MRI could enable a more complete characterization of RV and PA interactions in PAH. The purpose of this study was to non-invasively estimate PVR from TRV/QPA using a 4D flow-sensitive MRI sequence in humans [3].

## Methods

Six PAH patients referred for right heart catheterization (RHC) and six healthy volunteers were scanned according to an IRB-approved protocol. In patients, RHC was performed clinically within 1 week of the MRI in patients with PAH. PVRRHC was calculated as follows: PVR=ΔP/QPA. 4D flow MRI (Phase Contrast with Vastly undersampled Isotropic Projection Reconstruction - PC VIPR) was performed on 3T clinical scanners (GE Healthcare, Waukesha, WI) after the administration of gadolinium-based contrast agents. PC VIPR parameters: FOV=32 x 32 x 22 cm, isotropic 1.3 mm spatial resolution, TR/TE = 6.3/2.1 ms, Venc=150 cm/s, scan time: ~10 min using respiratory and retrospective ECG gating. Post-processing was done using Ensight (CEI, Apex, NC) and MatLab (The Mathworks, Natick, MA) to measure peak TRV and QPA. Statistical analysis included regression analysis to evaluate the correlation between TRV/QPA and PVRRHC. A regression equation was derived to calculate PVRMRI. Differences between PVRMRI and PVRRHC were assessed using Bland-Altman analysis. TRV/QPA ratios in healthy controls were compared to those in PAH patients using Student t-test.

## Results

Average (± standard deviation) PVRRHC was 7.15 ± 3.56 UW. The Pearson correlation coefficient between TRV/QPA and PVRRHC was 0.94. The equation derived from linear regression was PVRMRI = 0.69*PVRRHC - 2.84 UW. Using this equation, the average (± standard deviation) PVRMRI was 7.15 ± 3.35 (p=0.99). The mean difference between PVRMRI and PVRRHC was 0 with positive and negative levels of agreement of 2.4 and -2.4, respectively. Differences in TRV/QPA in healthy controls and PAH patients were statistically significant (p=0.0003).

## Conclusions

PVR can be accurately estimated noninvasively using 4D flow MRI. The results from this study indicate that 4D flow-sensitive MRI with PC VIPR can also be used to estimate PVR, complementing the analysis of alterations in flow patterns in the heart and pulmonary arteries in patients with cardiopulmonary disease. Data from a different cohort of patients will be used for validation.

## Funding

R01HL086939(N.C.C) and R01HL072260(O.W)

**Figure 1:**
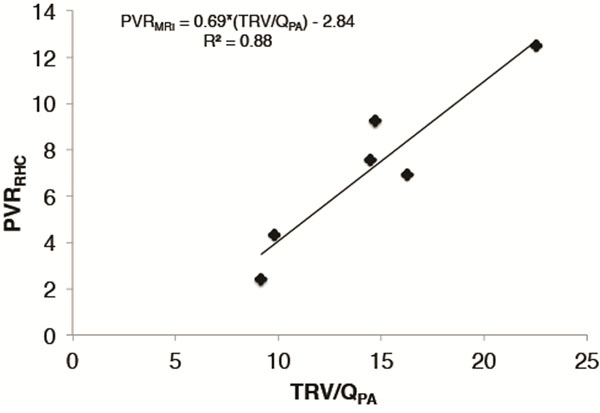
Scatter plot demonstrating relationship between TRV/QPA from 4D Flow (y axis) and PVR from RHC (x axis).

**Figure 2:**
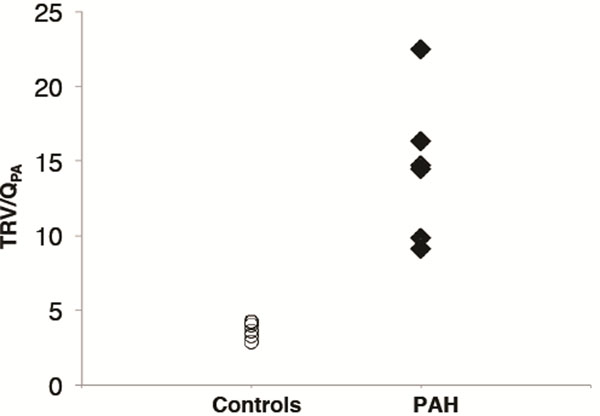
TRV/QPA comparison between PAH patients and healthy controls. Differences are statistically significant (p=0.0003)
